# Parental Perspectives of Sleep in the Home: Shaping Home–School Partnerships in School-Based Sleep Promotion Initiatives

**DOI:** 10.5888/pcd20.220395

**Published:** 2023-05-11

**Authors:** Melissa Bird, Kacey C. Neely, Genevieve Montemurro, Pamela Mellon, Megan MacNeil, Cary Brown, Lauren Sulz, Kate Storey

**Affiliations:** 1 Alberta Health Services, Calgary, Alberta, Canada; 2 Faculty of Health Sciences and Sport, University of Stirling, Stirling, United Kingdom; 3 School of Public Health, University of Alberta, Edmonton, Alberta, Canada; 4 Department of Occupational Therapy, Faculty of Rehabilitation Medicine, University of Alberta, Edmonton, Alberta, Canada; 5 Faculty of Education, University of Alberta, Edmonton, Alberta, Canada

## Abstract

**Introduction:**

Sleep is a critical component of child health and the prevention of chronic disease. Children may benefit from school-based sleep promotion; however, parents need to be involved for healthy sleep strategies learned at school to be translated to the home. The objective of this study was to explore parental perspectives on sleep behaviors and responsiveness to school-based sleep promotion.

**Methods:**

Twenty-five parents of school-aged children were purposively sampled for interviews from July 2019 through April 2020 in Alberta, Canada. Descriptive qualitative methodology was used, and data were generated through semistructured interviews and researcher field notes. Interviews were transcribed and themes were identified by using latent content analysis.

**Results:**

Three themes emerged from analysis: 1) sleep is valued and supported, 2) barriers to healthy sleep exist, and 3) schools are allies in promoting sleep. Parents perceived that sleep was essential for their child’s health, facilitated healthy sleep practices in the home, and highlighted barriers (busy schedules and poor parental role models) that affected sleep. Parents supported and expressed value in school-based sleep promotion and noted factors that affected the success of school-based sleep promotion.

**Conclusion:**

Parents are responsive to school-based sleep promotion. Promotion efforts should include resources that engage and involve parents in the school community. Throughout the development of resources to support school-based sleep promotion, additional consideration of parent-reported barriers to promoting healthy sleep in the home should be included.

**Summary**

**What is already known on this topic?**

Improving sleep behaviors among children promotes positive health outcomes. Schools are a critical setting where children learn healthy behaviors, which are translated to the home environment. How parents perceive school-based sleep promotion, and how they support the translation of learning at home, is not well understood.

**What is added by this report?**

Parents were responsive to school-based sleep promotion and valued a collaborative approach to support child sleep health. Parents cited barriers (busy lifestyle, parents as poor role models) to healthy sleep hygiene and school-based sleep promotion.

**What are the implications for public health practice?**

Findings support the implementation of school-based sleep promotion through strategies that can improve parent and school collaboration related to sleep.

## Introduction

Sleep is an essential component of a healthy lifestyle among children; however, declines in child sleep status warrant investigation and promotion of interventions (1). Without quality sleep, children are at risk of short- and long-term health consequences, including developing chronic diseases (1–7). Improving sleep practices through settings-based health promotion can promote health among children (8). Comprehensive school health, or its equivalencies (9), is an approach that prioritizes school, home, and community partnerships to reinforce healthy behaviors, including healthy sleep behaviors, across environments where children live, learn, and play (10). Although school-based interventions have been effective in improving healthy eating, active living, and academic outcomes (11), sleep has received minimal research attention (12). Parental participation is essential in sleep promotion; families substantially shape beliefs about sleep and can either reinforce or hinder healthy sleep practices at home (13–15). Whether parents support school-based sleep promotion and the translation of this knowledge to the home is unknown. Therefore, it is important to explore parents’ perceptions of sleep, their view on the role of the school in promoting sleep, and ways that parents facilitate or hinder their children’s sleep.

Engagement of parents in health promotion interventions aids in successful implementation (10,16). The comprehensive school health approach explores school-based sleep promotion, and home–school collaboration is a key component to its implementation (16). Such research will contribute to the field of school-based sleep promotion. Assessing parents’ knowledge about the importance of children’s sleep and their receptiveness to school-based sleep promotion allows for the informed development of strategies, ensuring that parental uptake is more likely, thus improving the likelihood of success. The objective of this research was to explore parental perspectives on sleep behaviors and how parenting practices facilitate and support or hinder their children’s healthy sleep behaviors.

## Methods

This study used a descriptive qualitative method. A method of naturalistic inquiry, the descriptive qualitative method aims to understand experiences, events, or processes in human experiences (17). Qualitative descriptive research has descriptive and interpretive validity because the researcher stays close to the data through summarization of participant experiences (18). This approach was used to explore parental perspectives of sleep behavior in the home to provide clear information to enhance school-based sleep promotion. Descriptions are in-depth narratives that help the reader understand a setting or situation. These rich descriptions can easily be understood by key community members, implementing partners, and knowledge users.

### Participants

Twenty-five parents of school-aged children (aged 5–12 y) were purposively sampled in Alberta, Canada. This project was part of a larger initiative aiming to improve how school-based sleep promotion programs could address sleep health in Alberta. We used maximum variation sampling, a form of purposeful sampling (differing geographic location and grade of students), to allow for phenomenal variation to represent diversity across the sample accurately (19). This process allowed for representation of parents from multiple school jurisdictions across the province with students in kindergarten through grade 6. Parental participation was not limited to those whose children had participated in a school-based sleep promotion program. Recruitment occurred as part of a larger project, Sleeping Soundly (https://www.katestorey.com/our-projects/sleeping-soundly), whereby information about this study was shared with parents through existing school connections and provincial partner networks, including APPLE Schools and Alberta Health Services. All participants who were recruited (N = 25) completed interviews. Recruitment methods included posters, newsletters, online postings, and word of mouth. Each parent read and signed the information letter and consent form before participating in the research project. Ethical approval was granted through the Human Research Ethics Board at the University of Alberta.

### Procedure

Each parent participated in a one-on-one semistructured interview in a public space or over the telephone. Interviews were conducted from July 2019 through April 2020. The interview guide was developed by University of Alberta researchers and other experts in school-based health promotion (Table). Interviews centered on parental perspectives and sleep practices in the home and the role of schools in promoting healthy sleep habits. Field notes and observations were also used in combination with interviews to contribute to the richness of the data and to align with a descriptive qualitative method.

**Table:** Interview Guide to Determine Parental Perspectives on Sleep Behaviors and Responsiveness to School-Based Sleep Promotion, Alberta, Canada, July 2019–April 2020

Topic	Questions
Parenting	How would you describe your overall approach to parenting?
	Do you parent differently in different situations?
	Has your parenting approach changed over the years?
	Do you parent different children in different ways?
Sleep	Describe the sleeping environment at home.
	Where do children and adults sleep? How many people in each bed? Are there pets in the room? Is there technology in the room?
	Do you feel that sleep is important for your child? Would you describe your child as a good sleeper?
	Are you concerned about your child’s sleep? If so, what concerns you?
	Do you ever seek out information to support sleep behaviors in your home environment? Where and who do you get this information from?
	Do you feel you could change your child’s sleep behaviors?
	How would you do this if you wanted to change your child’s sleep behaviors?
	Have you ever tried to change your child’s sleep behaviors? How did you do this?
	Describe any specific bedtime routines in your home.
	Explain any rules around your child’s sleep behaviors.
	In what ways does your sleep routine differ from that of your child?
	Do you believe that other health behaviors affect sleep?
School-based sleep education	What things does your child learn at school and share with your family at home? Has this impacted any changes in the home environment?
	Has your child ever shared information about any campaigns at school to promote healthy sleep with you? If so, did it impact any changes in your home?
	What role do you think your child’s school has in teaching healthy sleep behaviors?
	What role do schools play in teaching children about healthy sleep habits? Is this the responsibility of the school or the parents? Or a collaborative effort?
	In what ways do you think the whole family could use sleep education learned at school? What are some ways that this process can be improved?
	Are you aware that your child attends an APPLE School? Can you tell me about some of the initiatives that have occurred due to being in an APPLE School? (if applicable)
	What would an ideal bedtime routine look like for you and your child?
	What is the biggest barrier to a good night’s sleep?

### Data analysis

Interviews were audio-recorded and transcribed verbatim by a professional transcriptionist. Transcribed interviews and researcher field notes were imported into NVivo version 12 qualitative organizational software (QSR International). Data were analyzed after each parent interview, and initial thoughts and interpretations were recorded. Field notes provided essential information about the contextual environment of participants’ experiences and were incorporated into data analysis procedures. Latent content analysis was used to identify meaning units and to describe parental understanding of sleep behavior in the home and school-based sleep promotion. Transcripts were read and reread to identify meaning units and then assigned individual codes, each with descriptive inclusion criteria. Codes were then categorized and recategorized to reveal relationships within the data (20). Transcripts were read to ensure subcategories and codes were consistent with the theme meaning, and categories were separated when distinct ideas were identified. All data analysis was conducted by the researcher who was debriefed by a member of the research team throughout the analysis process to ensure accuracy of interpretations.

## Results

Parents resided in Northern Alberta (Edmonton, Peace River, Grande Prairie) and Southern Alberta (Calgary, Drumheller). All 25 participants were women; the average (SD) age was 39.0 (4.6) years. Their children were in grades kindergarten to grade 6; the average (SD) age was 8.3 (2.0) years. Parent-reported average child bedtime was 8 pm on weekdays and 8:30 pm on weekends. Average wake-up time was 6:45 am on weekdays and 7:15 am on weekends. Children’s sleep duration averaged 10.8 hours on weekdays and 10.6 hours on weekends.

The following themes emerged from the analysis: 1) sleep is valued and supported, 2) barriers to healthy sleep exist, and 3) schools are allies in promoting sleep. Parents perceived that sleep was essential for their child’s health and facilitated healthy sleep practices in the home but explained potential barriers that affected sleep in families.

### Theme 1: Sleep is valued and supported

Parents strongly emphasized the value of healthy sleep practices and described their experiences supporting their child’s sleep. Parents explained that sleep was important for their child to function in everyday life and believed that sleep was important for their child to do well in school and to manage their emotions.

**Subtheme: Parents recognize the importance of sleep.** Parents believed that their child’s sleep was important and illustrated how sleep had positive effects on their children. Sleep was perceived to affect children’s learning, attention, focus, and mood, and in turn, affected their child’s ability to function in school and in everyday life. One parent summarized this concept below:

Their brains are growing so fast they need to like, regroup and you know be able to absorb all that knowledge and grow, and be well rested, and I think it just affects everything. I think that sleep is just as important as eating healthy, right across the board they need proper sleep, cause if you don’t have it, you’re tired and you’re cranky. And then you can’t, you know, focus, perform well at school, or you just feel like crap. (Parent 47)

Parents observed that poor sleep affects a child’s ability to function and pay attention and voiced that well-rested children can focus better in school and pay attention to the concepts they are learning in class. Parents described their children as cranky, moody, quick to anger, and unable to regulate their emotions when they did not get enough sleep.

**Subtheme: Parenting practices support healthy sleep behaviors.** Parents identified practices that facilitated healthy sleep behaviors in their children. These practices included setting and enforcing rules and establishing a consistent bedtime routine. Parents indicated that setting and enforcing rules was crucial in developing healthy sleep practices for their children. As well, parents described that children who had clear rules and expectations understood what they should do when preparing for bed and could have more independence with their sleep routines. One parent used a Groclock (a sleep trainer clock for young children) to help their children understand when to go to bed and when to get up:

They have a Groclock in their room, so they have to stay in there — in their bedrooms until in the morning, until their Groclock comes up. Yeah, it works great, so yeah, that’s kind of — that’s the rule. Once they’re in bed they stay in bed until the clock goes off. (Parent 36)

Parents recalled that establishing a consistent bedtime routine helped to facilitate their children’s healthy sleep behaviors. One parent mentioned that to set their child up for success they needed to “have a routine . . . and consistency in what they do for the routine” (Parent 40). The bedtime routine was viewed as easier if their child could anticipate what their routine leading to bedtime would look like and would cue the children to relax and prepare for sleeping.

### Theme 2: Barriers to healthy sleep exist

Although parents valued sleep behaviors in the home and carried out practices to support sleep in their children, they described lifestyle factors that negatively contributed to healthy sleep hygiene in the home. These lifestyle factors were described as having a busy lifestyle and not prioritizing sleep as a parent.

**Subtheme: Busy lifestyle.** Parents described that there are “times where we’re just go, go, go, and we’ve been so busy in the evening” (Parent 13), and this busy schedule caused their child(ren) to go to bed at a later time. Parents indicated that it is “chaotic when there are [commitments] after school, and by the time you get home, have dinner, it’s just a little bit later” (Parent 52). Having a busy schedule was a barrier to establishing a routine sleep behavior in children because it tends to keep children up later.

**Subtheme: Parents as poor role models.** Parental lifestyle was recognized as a barrier to healthy sleep habits in the home. Parents described that their sleep practices were often very different from their children’s. One parent recalled that: “We’re probably very bad role models. We tend to stay up later and wake up at the last moment” (Parent 31). One parent indicated that parents do not set a good example to their children and described the effect this may have on their children’s sleep behavior:

There’s not one person that I know that’s a parent that sets a good example of sleep. The fact that kids they do what they see, as opposed to what they hear. So unless they actually understand why they need sleep, and unless they can actually understand the difference that makes in their own life and see it themselves. They may not always listen, and it may be harder for them to get to sleep. (Parent 20)

Parents also felt that they did not have “a good routine going to sleep” and were “always so busy getting stuff done” (Parent 28) that they do not have time to wind down and have a sleep routine. Many parents cited having a television in their room was negative role modeling. Parents indicated that they knew “a lot of adults don’t follow the ‘no technology rule’ before bed” (Parent 13).

### Theme 3: Schools are allies in promoting sleep

Parents viewed sleep promotion in schools as a valuable tool to reach children and provide education on sleep and believed that children could learn about sleep in school and transfer this knowledge to their family members in the home. However, parents believed that school-based sleep promotion initiatives required support from both the school and the home to be successful.

**Subtheme: Sleep promotion in school is valuable to reach children.** Parents felt that sleep promotion in schools was valuable and could help children learn how to improve their sleep. Parents described that learning about sleep in school could become “a habit that would be good and healthy for them” (Parent 22). Parents thought that if students were not “learning about [sleep] at home, then school would be a good place for them to hear it” (Parent 39). One parent noted how sleep promotion in school is important to support families and the community:

But when you have parental and family structures like we do and we’re seeing more and more, with double working families, or things like that — the community, especially the school has a bigger role to play. . . . I think if we’re putting things like sleep or nutrition, these things as part of our health unit, it’s really important. (Parent 16)

One parent mentioned their view on the importance of integrating sleep into the classroom as a mechanism to reach more children:

I think it should kind of be something that gets mentioned, I mean they have health classes and stuff like that, so I think it’s definitely something that they can discuss in school ’cause not all parents are going to discuss that with their kids. . . . I know that a lot of kids don’t get that from their parents, so I know that if the school were to help out a little bit teaching that, I think that it would — it would be very good. (Parent 52)

**Subtheme: Sleep promotion at school can translate to the home.** Parents believed that students who learned about sleep at school could bring this information home and foster healthier sleep habits in the home. Parents noted that sleep promotion in schools would help reinforce the importance of sleep. Parents suggested that students could then bring awareness of healthy sleep practices back to the home and engage the family:

I think, just school’s role in having those discussions, and setting that value for sleep, can help, right, can help students. If teachers are saying how important it is, and things like that, maybe that helps bring that conversation out to the home, so that their awareness is better that every — every — it’s important for everyone and everyone’s doing it. (Parent 31)

Parents needed to support their children to achieve healthier sleep behaviors in partnership with the school:

I mean it’s on both. We’ve had this conversation at work where other people are like, “Oh, they’re not learning that in school. Well then, it’s the parents job to teach them some of these like life skills.” Right, so, I think it’s both. I mean it needs to be coming from both sources, parents — parents need to sort of reinforce maybe what’s coming home from school — but I mean it’s both school and parents. (Parent 44)

## Discussion

Parents valued and supported healthy sleep practices, recognized barriers to healthy sleep in the home, and identified schools as allies in promoting sleep. Parents shape their children’s sleep practices in the home from a young age and continue to influence their children through their elementary years (21). Parenting practices and parent–child relationships affect child sleep (22), while differing views of problematic sleep behavior (23) may cause chronically inadequate sleep and health consequences. In our study, parents demonstrated an accurate knowledge of the effect of inadequate sleep on their children. Parents appeared to be aware of the potentially harmful effects of inadequate sleep on their children and were motivated to improve their children’s sleep behaviors. Parents reported that they thought sleep was valuable for their children’s learning, development, attention, focus, energy, mood, academic achievement, and coping and resiliency; these outcomes have been substantiated in the literature (24). Overall, parents were knowledgeable of the effect of inadequate sleep on their children.

Parents cited barriers to achieving healthy sleep habits in the home and reported that their family’s busy lifestyle limited their ability to establish a consistent bedtime routine and bed/wake times. Both extracurricular commitments and a busy schedule were previously identified as barriers to child sleep (25). Our study showed that parents recognize themselves as poor role models for healthy sleep behavior. Although some literature indicates the importance of parents as role models for physical activity (26) and healthy eating (27), the effect of parent role modeling on sleep practices is not strongly demonstrated. However, one study suggested that parents may decrease their children’s screen time by reducing their own screen times (28). Because screen time use before bed is negatively correlated with a child’s sleep quality (29), parents may indirectly improve their child’s sleep behavior through role modeling by reducing their own screen time. Additional research is needed to explore the effect of parent role modeling on their children’s healthy sleep practices and investigate viable strategies to address parent-reported barriers to healthy sleep habits.

Since this research was conducted, the Canadian Sleep and Circadian Network released a national strategy to integrate sleep research priorities and policy into public health and identified school-based sleep promotion as a target (30). However, exploration and evidence of parent receptivity to school-based sleep promotion are limited. Parents have been found to support school policy changes that promote healthy eating habits (31). Studies that evaluate parent perspectives of comprehensive school health interventions have not included sleep and tended to focus on physical activity, nutrition, and sedentary behavior, with most parents viewing that these behaviors are a collective responsibility of both the school and home (32). Thus, our study provides new insight: parents are supportive of school-based sleep promotion, are willing to be involved in school-based sleep promotion, and view it as a strategy to reach families with diverse needs who may not promote sleep at home due to social and economic influences. It is promising that parents believed their children would share information at home and cause changes in the family. This study demonstrates that parents view school-based sleep promotion as a viable option for improving child sleep and are supportive of their children’s school-based learning about sleep health. Thus, it is likely that school-based sleep promotion interventions will be successful.

### Limitations and strengths

This study has several limitations. First, all of the participants identified as women. It is well established that mothers influence child sleep, but other family members (eg, fathers, grandparents, siblings, guardians) and caregivers can also influence sleep behaviors. Second, 8 of the 25 parents were interviewed during the early onset of the COVID-19 pandemic. Lifestyle changes caused by the pandemic may have shifted their attitudes toward child sleep, and their responses may have been different had they been interviewed before the pandemic. Third, researchers used a Westernized lens and promoted sleep practice guidelines that best suit a Eurocentric viewpoint. The diverse cultural heterogeneity in the Canadian population demonstrates that various sociopolitical and environmental factors (eg, socioeconomic status, race and ethnicity, sex and gender, cultural and family traditions) affect sleep, and some perceptions of sleep may not be fully represented by this research project. A strength of this research was the purposeful sampling of parents to include participants from across multiple jurisdictions in Alberta, which allowed for diversity across the sample.

### Conclusions

This research was conducted to explore parent responsiveness to school-based sleep promotion that uses a comprehensive school health approach. This study found that participating parents were well-informed of the importance of adequate sleep in their children and perceived that sleep was valuable and supported in the home. Parents viewed school-based sleep promotion as a strategy to promote awareness of healthy sleep among children who may not learn about sleep at home. Parents recognized that their own sleep behaviors affected the promotion of healthy sleep in the home. A range of behavioral change strategies is needed to address sleep on various socioecologic levels. For example, parents noted the effect of social and economic influences on health and noted that schools can be environments to address health inequalities. Grounded in the comprehensive school health approach, the school is an organizational component that can address individual and interpersonal factors. This research underscores the importance of home–school partnerships in promoting children’s sleep. It is recommended that future school-based sleep promotion include options that support families with diverse needs, including free or low-cost pathways to health supports and services. In elementary grade levels, educators can incorporate lesson plans that actively engage the whole family, such as interactive games or home sleep challenges to raise awareness of the importance of sleep in the home. Additional consideration of parent-reported barriers to promoting healthy sleep in the home is required in future school-based programs for tailored, effective sleep promotion. Overall, the results of this study demonstrate that parents view school-based sleep promotion as a viable option for improving child sleep. Additional research should explore educator, administrative, and division-level perspectives in implementing school-based sleep promotion that uses a comprehensive school health approach.
